# Supplementary material for: Incarcerated individuals and education programmes in Nigeria: A task for social workers
Source: PLoS One. 2023 Jul 20;18(7):e0288693. doi: 10.1371/journal.pone.0288693 (PMC10358994; doi:10.1371/journal.pone.0288693)
Supplement: Attachment — Submitted filename: A point-by-point response to reviewers.pdf [file pone.0288693.s002.pdf]

RESPONSE TO REVIEWER'S COMMENTS

Hull York Medical School
University of Hull
Hull, United Kingdom
February 16, 2023

The Editor,
PLOS ONE.

Dear Dr Johannes Stortz,

A point-by-point response to the editor's and reviewer's comments

Please find below our response to the points raised by the reviewer.

Best wishes,

Blessing O. Ukoha-Kalu; PhD

S/N	Comments	Response
Editor's comments		
1.	Please ensure that your manuscript meets PLOS ONE's style requirements	Thank you for your comment. We have now ensured that our manuscript meets PLOS ONE's style requirements.
2.	Please provide additional information regarding the considerations made for the prisoners included in this study. For instance, please discuss whether participants were able to opt out of the study and whether individuals who did not participate receive the same treatment offered to participants.	Thank you for your comment. We have now provided additional information regarding the considerations made for the prisoners included in the study. See page 7, lines 141-142; page 7-8, lines 160-162
3.	Your ethics statement should only appear in the Methods section of your manuscript. If your ethics statement is written in any section besides the Methods, please delete it from any other section	Thank you for your comment. We have now included the ethics statement in the methods section only
Reviewer's comments		
1.	However, I think there is currently a mismatch between research questions, methods used and conclusions	Thank you for your comment. We have now addressed this. See lines 113- 114
2.	The research questions are too broad and not all	Thank you for your

	suitable for answering with interview data on 20 individuals. The authors formulate the questions as follows: The objectives of the study include 1) Education programs available for inmates. (2) Inmates' perception and essence of correctional education. (3) The quality of correctional education programmes and inmates' participation. (4) Social workers and correctional education.<br>I think the manuscript would benefit from formulating one or two more specific questions. This would also enable/create room for a more in-depth analysis of the interview data.	comment. This study has been concluded. However, we have identified this as one of the study's limitations. See page 20, lines 445-447
3.	Another recommendation would be to more critical of the effects of educational programs and the effects of social work in a correctional setting. The authors mention this sometimes but the conclusions are still very positive about the expected effects of educational programs- even though this question is not examined in the current study and prior research shows very mixed effects.	Thank you for your comment. We have now added another recommendation on the need for social workers in a correctional setting. See page 20-21, lines 464-466
4.	In general, the authors draw several conclusions that do not directly follow from their study. For example, the statement 'Employment should be given to ex-inmates…' requires further explanation.	Thank you for your comment. We have now rewritten the conclusions to reflect the study objectives. See lines 450 - 457
5.	In recent years it has become more common to use terms as incarcerated individuals instead of inmates (thereby acknowledging the individual is more than his/her crime).	Thank you for your comment. We have now replaced 'inmates' with 'incarcerated individuals in the manuscript.
