# Supplementary material for: Incarcerated individuals and education programmes in Nigeria: A task for social workers
Source: PLoS One. 2023 Jul 20;18(7):e0288693. doi: 10.1371/journal.pone.0288693 (PMC10358994; doi:10.1371/journal.pone.0288693)
Supplement: Attachment — Submitted filename: RESPONSE TO REVIEWERS COMMENTS.pdf [file pone.0288693.s003.pdf]

School of Medicine,
University of Nottingham,
Nottingham, England,
United Kingdom
June 26, 2023

The Editor,
PLOS ONE.

Dear Editor,

A point-by-point response to reviewer's comments

Please find below our response to the points raised by the reviewer.

Best wishes,

Dr Blessing Onyinye Ukoha-Kalu

Title: Incarcerated individuals and education programmes in Nigeria: A task for social workers

**Comment**	**Response**
**Weakness**	
In both the introduction and results, the authors offered the same frameworks as the objectives of the study and four themes. However, the reviewer thinks that the authors should propose more specific research questions in the introduction to assist readers to understand the hypothesis of this research. The reviewer understood that the authors recognized this issue as a limitation of research. However, the reviewer strongly advises the authors to focus on research questions connected to the research results to make it more established as a research paper.	Thank you for your comment. We have now proposed more specific research questions in the introduction that will help the readers to understand the hypothesis of this research.<br><br>See line 105-112; 344
In methods, the authors should provide exact information on four questions for readers to understand what kind of questions were asked. It is important to explain the relationships between the four questions and	Thank you for your comment. We have now provided exact information on the four questions and also explained the relationships between

the four themes of results.	the four questions and the four themes identified.<br><br>See lines 180-189, 200
In the results, the authors should include the genders of the participants	Thank you for your comment. We have now included the genders of the participants.<br><br>See Line 204 – Table 1
In Table 1, the authors should double-check if personal information is identifiable. To protect personal information, the authors could provide statistical information instead of individual data.	Thank you for your comments. The authors used pseudonyms throughout. See lines 166 – 167. The personal information used is not identifiable at all.
In terms of female incarcerated individuals, the authors stressed the advantage of Owerri prison including females in the introduction, but not much discussed focusing on genders. The reviewer understood the limitation of the research due to the few female candidates, but the authors could propose some discussion, depending on the female interviewees. The reviewer advises the authors to provide some ideas or recommendations from the viewpoint of gender for future practices.	Thank you for your comment. We have now provided recommendations from the viewpoint of gender for future practices.<br><br>See lines 479-480
**Some minor points**	
Some remain as inmates. Please correct all inmates to incarcerated individuals	Thank you for your comments. We have now made the correction.<br><br>See lines 144, 155, 206, 228, 229, 251, 263, 271, 280, 298, 299, 318, 333, 356, 357, 253, 266, 320
In the sampling procedure, the authors insisted that they recruited older individuals 3 years of service and above, but in the Table 1, some participants served less than 3 years. The authors should double-check the condition of sampling	Thank you for your comment. We have double-checked and made the correction.<br><br>See Table 1
