# Supplementary material for: Incarcerated individuals and education programmes in Nigeria: A task for social workers
Source: PLoS One. 2023 Jul 20;18(7):e0288693. doi: 10.1371/journal.pone.0288693 (PMC10358994; doi:10.1371/journal.pone.0288693)
Supplement: Attachment — Submitted filename: Response to academic editors comments.docx [file pone.0288693.s001.docx]

Hull York Medical School

University of Hull

Hull, United Kingdom

October 25, 2022.

The editor,

PLOS ONE.

Dear Sir,

Please find below my response to the academic editor’s comments on our manuscript titled “**Prison inmates and education programs in Nigeria: A task for social workers”**

Thanks for your consideration of this manuscript.

Best wishes,

Blessing Ukoha-Kalu

| Comment | Response |
| --- | --- |
| Firstly, the connection between prison education and the role of the social worker in implementing such a programme is unclear. This connection needs to be made clear throughout the manuscript. | We have addressed this point and amended the ‘manuscript’. Pages 2 and 4 |
| Secondly, the analysis of theme 1 on the availability of the education programme needs to be supported by the data. | We have addressed this point and amended the ‘manuscript’. Pages 9-10 |
| Finally, some sections require more than just one quote to demonstrate the rigour and trustworthiness of the study. | We have addressed this point and amended the ‘manuscript’. Pages 10-15 |
